# Non-contrast enhanced, EKG-triggered, navigator MR angiography of the thoracic aorta and proximal pulmonary arteries: initial evaluation of using an abdominal compression band to reduce acquisition times

**DOI:** 10.1186/1532-429X-16-S1-P375

**Published:** 2014-01-16

**Authors:** Emer Sonnex, Indrajeet Das, Richard Coulden

**Affiliations:** 1 Dept Radiology & Diagnostic Imaging, University of Alberta Hospital, Edmonton, Alberta, Canada

## Background

Non-contrast enhanced, EKG-triggered, navigator 3D-SSFP magnetic resonance angiography (NCE-MRA) is a robust technique but suffers from long scan acquisition times. Depending on heart and respiratory rates, acquisition times can still be prohibitively long. We describe the use of an abdominal compression band to reduce respiratory motion and image acquisition time with no sacrifice of image quality.

## Methods

Ethics committee approval and informed consent were obtained. 20 normal volunteers (mean age: 39) underwent two NCE-MRA examinations. Both examinations were performed free-breathing, one with an abdominal compression band (band) and one without (no-band). Both examinations were performed on the same 1.5T Aera (Siemens Medical Solutions) with identical scanning parameters within 10 minutes of each other. Each examination duration was recorded. All angiographic data sets were anonymized. Data sets were reviewed in random order by three cardiothoracic radiologists on a single workstation. Motion artifact and overall quality were assessed semi-quantitatively on a scale of 1 - 4 (excellent/no artifact - 1; good/minor artifact/noise - 2; moderate/some noise/artifact - 3; poor/limited diagnostic quality - 4).

## Results

NCE-MRA was completed successfully with and without an abdominal compression band in all volunteers (40 data sets). There was no difference in global image quality or motion artifact at the aortic root between 'band' and 'no-band' examinations (mean image quality 1.31 and 1.51; mean aortic root artifact 1.25 and 1.44 respectively). No studies in either group were rated moderate or poor. None of the observers could reliably distinguish between 'band' or 'no-band' examinations. Scan times between the 2 groups were, however, significantly different. Scan times ranged from 4.5 - 9.1 minutes (mean 6.7; SD 1.14) in the 'no-band' group and 3.3 to 8.9 minutes (mean 5.0; SD 1.3) in the 'band' group. Mean reduction in acquisition time between the two groups was 1.7 mins or 25% of the standard technique (p = 0.0025). Only 1 data acquisition time increased with the abdominal band.

## Conclusions

Using an abdominal compression band in non-contrast enhanced, EKG-triggered, navigator 3D-SSFP MRA significantly reduces image acquisition time (25%) without sacrificing image quality. This technique has only been used in volunteers to date. Further study will be needed to see if this benefit is maintained in patients.

## Funding

None.

**Figure 1:**
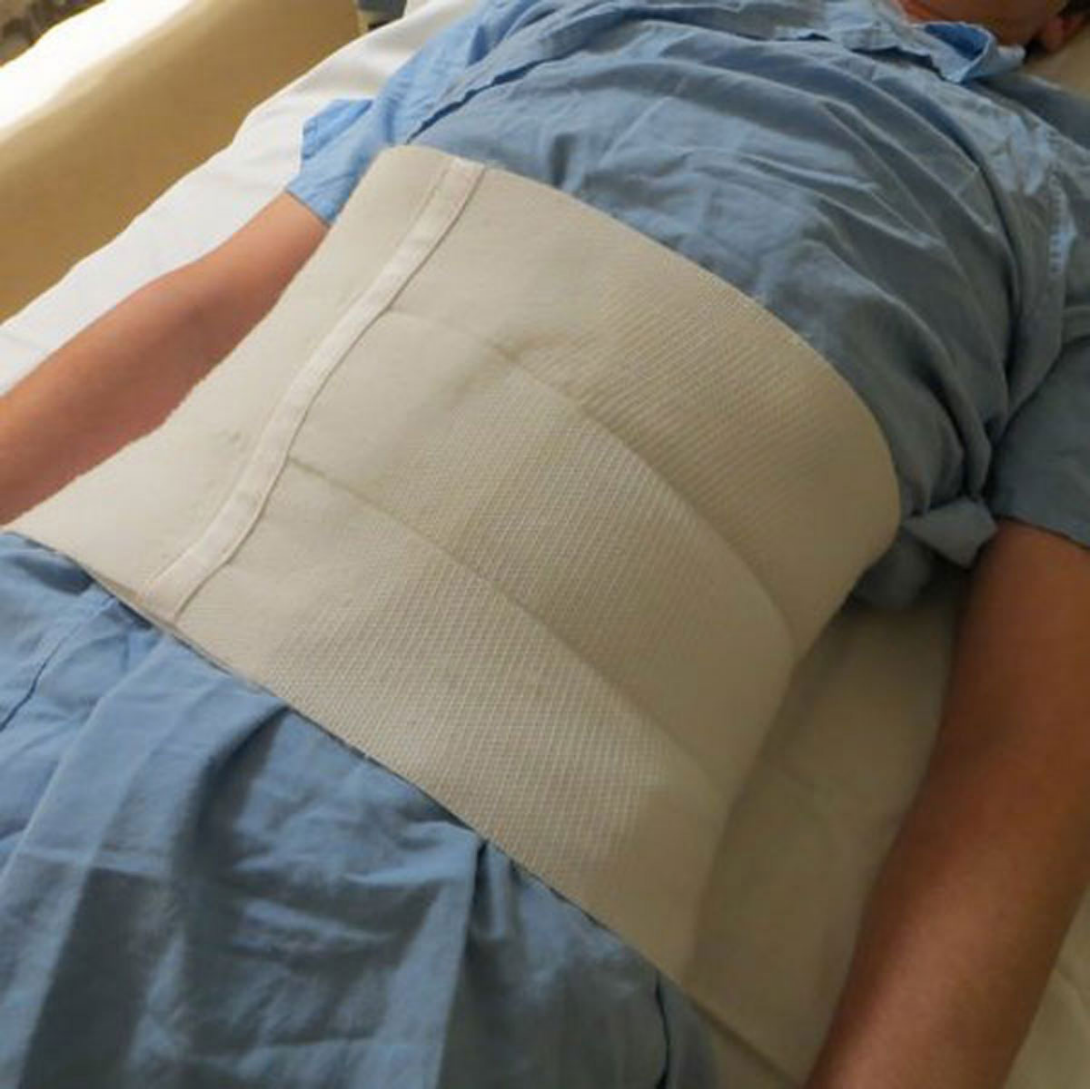
**Broad, elasticated abdominal belt applied tightly at end expiration**.

